# Human Induced Pluripotent Stem Cell-Derived Brain Endothelial Cells: Current Controversies

**DOI:** 10.3389/fphys.2021.642812

**Published:** 2021-03-31

**Authors:** Tyler M. Lu, José Gabriel Barcia Durán, Sean Houghton, Shahin Rafii, David Redmond, Raphaël Lis

**Affiliations:** ^1^Division of Regenerative Medicine, Department of Medicine, Ansary Stem Cell Institute, Weill Cornell Medicine, New York, NY, United States; ^2^Ronald O. Perelman and Claudia Cohen Center for Reproductive Medicine, Weill Cornell Medicine, New York, NY, United States

**Keywords:** induced pluripotent stem cells, endothelial cell, epithelial cell, cell fate and differentiation, misclassification, brain–blood barrier (BBB), disease modeling

## Abstract

Brain microvascular endothelial cells (BMECs) possess unique properties that are crucial for many functions of the blood-brain-barrier (BBB) including maintenance of brain homeostasis and regulation of interactions between the brain and immune system. The generation of a pure population of putative brain microvascular endothelial cells from human pluripotent stem cell sources (iBMECs) has been described to meet the need for reliable and reproducible brain endothelial cells *in vitro*. Human pluripotent stem cells (hPSCs), embryonic or induced, can be differentiated into large quantities of specialized cells in order to study development and model disease. These hPSC-derived iBMECs display endothelial-like properties, such as tube formation and low-density lipoprotein uptake, high transendothelial electrical resistance (TEER), and barrier-like efflux transporter activities. Over time, the *de novo* generation of an organotypic endothelial cell from hPSCs has aroused controversies. This perspective article highlights the developments made in the field of hPSC derived brain endothelial cells as well as where experimental data are lacking, and what concerns have emerged since their initial description.

## Introduction

The latest efforts to develop drugs targeting neurodegeneration and neurological disorders have been met with disappointment in recent clinical trials. The relative ineffectiveness of those drugs has incited the scientific community to develop better pre-clinical models by improving human cell-based models to capture the complexity of the brain. While the discovery of induced pluripotent stem cells and subsequent generation of brain organoids has advanced innovative avenues, these brain organoids are still rudimentary, lacking primordial non-neuronal cell types of the central nervous system (CNS) like microglia and most importantly functional blood vessels. During embryonic development, endothelial cells (ECs) acquire unique organ-specific molecular and cellular specializations that are crucial for the formation of the blood-brain-barrier (BBB) and therefore the maintenance of brain homeostasis. Human pluripotent stem cells (hPSCs), embryonic or induced, have been used in an effort to generate large quantities of specialized cells for development studies and disease modeling. The use of hPSCs to generate a pure population of these specialized brain microvascular ECs (iBMECs) has been described to meet the need for a reliable and reproducible *in vitro* BBB model. Specifically, it has been reported that hPSC-derived iBMECs display EC-like properties including tube formation, low density lipoprotein uptake, high transendothelial electrical resistance (TEER), and select barrier-like transporter activity. Over time, this *de novo* generation of an organotypic endothelial cell from hPSCs has aroused controversies. This perspective article highlights the developments made in the field of hPSC derived brain endothelial cells as well as where experimental data are lacking, and what concerns have emerged since their initial description.

## From Generic to CNS Specific Endothelial Cells

Brain endothelial cells plays an essential role in the development of a multicellular vascular structure separating the central nervous system (CNS) from the peripheral blood circulation (Bär, 1980; Risau and Wolburg, 1990; Engelhardt, 2003). In mammals, this process begins when cells originating from the mesoderm, known as angioblasts, enter the head region and form the perineural vascular plexus (PNVP) which will go on to encompass the neural tube by mid-gestation. None of the microvasculature in the CNS is derived from the neuroectoderm but instead, new vessels sprout from the existing PNVP into the developing neuroectoderm (Risau and Wolburg, 1990; Engelhardt, 2003). This process is highly regulated, occurring at precise stages of embryonic development, thus leading to the formation of a reproducible pattern of neuro-vasculature in all mammals (Aird, 2007a, b).

Initial signs for CNS angiogenesis and induction of BBB traits are given by the neural microenvironment on embryonic day E10 in mice (Obermeier et al., 2013). Endothelial cell progenitors from the PNVP infiltrate the neuroectoderm following a gradient of vascular endothelial growth factor (VEGF), resulting in the development of nascent “leaky” or “immature” blood vessels (Potente et al., 2011). Activation of the Wnt/β-catenin pathway in these nascent blood vessels triggers the expression of genes critical for the formation of the BBB. Wnt ligands secreted by the neural microenvironment bind to a set of receptors expressed by the endothelial cells (Frizzled, LRP5, LRP6) to elicit the expression of GLUT1, DR6, and TROY (Stenman et al., 2008; Tam et al., 2012). Furthermore, G-protein coupled receptor 124 (GPR124) seems to be essential for barrier genesis in the brain as it acts as an endothelial specific co-activator of Wnt/β-catenin signaling in the BBB (Kuhnert et al., 2010; Anderson et al., 2011; Cullen et al., 2011). By day E15 an embryonic BBB is formed in mice (Daneman et al., 2010; Ben-Zvi et al., 2014); however, the exact timing of BBB formation in human development and whether humans are born with a fully mature BBB remains unclear (Saunders et al., 2013).

This primitive BBB further mature by recruiting pericytes to the developing blood vessels. This step is critical to ensure proper BBB formation and function (Armulik et al., 2010; Bell et al., 2010; Daneman et al., 2010; Vanlandewijck et al., 2018). A recent study deconvoluted the complexity of the endothelial responses to pericytes at the single cell level (Andaloussi Mae et al., 2020). Activation of endothelial TIE2 signaling by ANGPT2 secreted by pericytes reinforce endothelial arterio-venous zonation, angiogenic quiescence and a limited set of BBB functions. It was also shown that the last component of the BBB, astrocytes, support the endothelial cells in acquiring BBB attributes and barrier properties (Alvarez et al., 2011).

## Organotypic Properties of Brain Microvascular Endothelial Cells

The major endothelial transport systems, ions channels and GPCRs are described in detail elsewhere (Daneman and Prat, 2015; Vanlandewijck et al., 2018; Hariharan et al., 2020). The unique cellular junction molecules expressed by brain endothelial cells are briefly discussed below.

The BBB is lined with specialized endothelial cells (EC) known as brain microvascular endothelial cells (BMEC) that possess intercellular tight junctions (TJs), lack fenestrations, and greatly limit transcytosis. BMECs, acting in conjunction with various neural cell types and non-cellular elements, form the BBB which regulates the dynamic transfer of select molecules into and out of the CNS (Zlokovic, 2008; Daneman et al., 2009; Daneman, 2012). These properties are achieved through the presence of distinctive TJs exhibiting a high *trans*-endothelial electrical resistance (TEER) *in vivo* and reduced caveolar-mediated transport, along with the presence of selective transporters. Due to their significant structural and functional overlap, most of the current understanding of endothelial cell TJs has been derived from examination of their epithelial counterparts (MDCK, CACO2, and ECV304) (Garberg et al., 2005). However, BBB-endothelial TJs hold many unique attributes which may be more akin to ECs of other organs when paracellular permeability and dynamic regulation are evaluated under pathophysiological conditions.

The establishment and maintenance of BBB TJs are governed by mainly three transmembrane proteins: Claudins, Occludin, and Junction Adhesion Proteins (JAM). The Claudin family is comprised of at least 24 member proteins which contain two extracellular loops responsible for homophilic interaction as well as establishing a link with claudins of contiguous endothelial cells. This homophilic interaction forms the primary seal of the TJ *in vivo* (Piontek et al., 2008) with Claudin -1, -3, -5, and -12 initially thought to be expressed by BBB-forming ECs. While some studies showed immunostaining against Claudin-1 at the BBB in rodent models (Liebner et al., 2000), it has since been shown that it is not expressed by BBB-forming ECs (Pfeiffer et al., 2011). Likewise, while some research groups have reported that brain microvasculature expresses Claudin-3 (Wolburg et al., 2003), others could not reproduce or confirm this observation (Kominsky et al., 2007; Steinemann et al., 2016). The generation of a Claudin-3^–/–^ mice demonstrated that the junctional immunostaining produced by anti-Claudin-3 antibodies in mouse brain ECs *in situ* and *in vitro* is not due to the presence of Claudin-3 but rather to an endothelial junctional antigen that is still present in brain ECs of Claudin-3^–/–^ mice (Castro Dias et al., 2019). Of note, it is now known that Claudin-1 and -3 are selectively expressed by the epithelium of the choroid plexus (Steinemann et al., 2016). Thus, these contradictory observations emphasize the contentious reliability of assessing Claudin protein expression in BBB TJs though their transcript expression still remain an important measure of cell specific tight junctions.

Another barrier property of the BBB lies in their ability to restrict immune cell infiltration to the CNS as BMECs generally has a low expression of leucocyte adhesion molecules during homeostatic conditions (Reese and Karnovsky, 1967; Brightman and Reese, 1969; Westergaard and Brightman, 1973). Immune cell trafficking across the BBB during pathophysiological conditions have been extensively studied in animal models of neuroinflammatory disease. These studies highlight the unique interaction of immune cells with BMECs which is regulated by a sequential cascade of different signaling pathways involving various adhesion molecules. BMECs harbor unique intrinsic properties which allow them to adapt and respond to inflammatory cues and thereby regulate immune cell trafficking through the BBB. BMECs exposed to TNFα, IL-1β, and IL-6 have shown increased paracellular permeability as well as acquiring an activated phenotype (de Vries et al., 1996).

These phenotypical modifications, unique to the vascular cells, are mainly characterized by an induced expression of endothelial cellular adhesion molecules that are critical for the recruitment of circulating leukocytes to sites of inflammation. The importance of ICAM-1 in regulating leukocyte recruitment during neuroinflammation has been highlighted in different animal models using both ICAM-1-null mice and ICAM-1-blocking antibodies (Zhang Rui et al., 1995; Kitagawa et al., 1998). An *in vivo* study in mice showed that E-selectin deficiency exert a neuroprotective effect characterized by reduced inflammation and neuronal apoptosis (Ma et al., 2012). Additionally, genetic P-selectin knock-in mice show increased BBB permeability and stroke injury (Kisucka et al., 2009). Hence, any BMEC cultured *in vitro* must be able to phenocopy this response to inflammatory stimuli in order to be considered physiologically relevant BBB model.

## A Brief Overview of *In Vitro* Blood Brain Barrier Models

The development of *in vitro* models has accelerated mechanical studies on the BBB as well as large scale screening of drugs with potential to penetrate the brain, with some limitations. Many studies have been conducted using primary BMEC isolated from various animal tissues, most commonly bovine, porcine, and rodent (Dehouck et al., 1990; Gaillard et al., 2001; Deli et al., 2005; Roux and Couraud, 2005; Zhang et al., 2006; Burek et al., 2012; Yusof et al., 2014; Helms et al., 2016; Veszelka et al., 2018). BMECs isolated from larger animal models generally possess higher TEER, at around 800 Ω.cm^2^ (Rubin et al., 1991), and low permeability due to high expression of junctional markers such as claudin-5, ZO-1, and occludin (Rubin et al., 1991; Cecchelli et al., 2007; Cohen-Kashi Malina et al., 2009; Patabendige et al., 2013). In particular, bovine and porcine brain ECs can be isolated in large quantities with ease; as a result, they have become the preferred choice for many permeability and transcytosis studies. Rodent brain ECs, specifically mouse or rat, have also been widely used as an *in vitro* model of the BBB with some groups developing immortalized cell lines (Roux et al., 1994; Wagner and Risau, 1994; Burek et al., 2012) and others discovering the use of Puromycin to increase the purity of primary isolations (Perrière et al., 2005; Calabria et al., 2006). Brain ECs these models generally possess lower TEER (under 300 Ω.cm^2^) (Daneman et al., 2010) but offer an avenue for BBB studies in transgenic models. Models using rodent brain ECs also provide the opportunity for large cohort studies and cells which can be targeted by many established antibodies.

These animal BMECs have also been studied in many co-culture conditions allowing for the discovery of many important cellular interactions between BMECs, astrocytes, and pericytes in the neurovascular microenvironment (Gaillard et al., 2001; Coisne et al., 2005; Garberg et al., 2005; Nakagawa et al., 2007; Helms et al., 2010, 2012; Liu et al., 2014). These co-culture models also possess higher TEER with the larger animal models exceeding 2,500 Ω.cm^2^ in some studies (Helms et al., 2014). Over time, BBB models developed using these various animal cell lines have demonstrated well-characterized permeability phenotypes and physiological similarities to human BMECs (Warren et al., 2009; Shawahna et al., 2011; Uchida et al., 2011; Hoshi et al., 2013). For instance, bovine co-culture models possess highly differentiated junctions which allow for various permeability and junction modulation assays using drug compounds (Wolburg et al., 1994; Gaillard and de Boer, 2000; Schaddelee et al., 2003; Boveri et al., 2006; Bohara et al., 2014). Rodent co-culture conditions utilizing single or multiple neural cell types have been shown to successfully mimic the neurovascular unit and even induce certain BBB phenotypes such as elevated TEER *in vitro* which has been validated by small molecule permeability screening (Coisne et al., 2005; Nakagawa et al., 2007, 2009; Abbott et al., 2012; Watson et al., 2013). Animal BBB models have provided a wealth of insight into various aspects of BBB physiology and pathology with a large amount of cross-validation between models. However, recent advances in the field have illuminated aspects in which animal models are lacking such as precise reproducibility with certain models showing a wide range of varied TEER and junctional phenotypes between laboratories (Schaddelee et al., 2003; Helms et al., 2014).

In an effort to generate a completely homologous model for clinical research and drug development, human primary BMECs have also been used in *in vitro* BBB models but are difficult to procure in sufficient numbers for experimental purposes (Bernas et al., 2010). Though human BMECs have provided a useful model for the study of many developmental and regulatory neurovascular pathways, the ethical questions and general restrictions placed on obtaining healthy human brain tissue along with low BMEC yields during isolation places a substantial limitation on their use in *in vitro* studies (Bernas et al., 2010). In addition, often times human BMECs offered by commercial vendors lack detailed documentation as to the isolation and sourcing of the cells, creating concerns over their use in many physiological models (Helms et al., 2016). The barrier properties and endothelial identity of primary BMECs are also not well maintained *in vitro* for extended periods of time, rendering them suboptimal for a number of potential BBB assays (Helms et al., 2016).

In order to overcome these limitations, immortalized human BMEC lines were established (Stins et al., 2001; Weksler et al., 2005; Rahman et al., 2016). These cells provided researchers with a model of human BMECs which was easy to use and had less batch variation availability issues. However, immortalized human BMECs also lose many of their brain specific EC attributes and produce a sub-physiologic TEER *in vitro* making them ineffective for functional studies (Urich et al., 2012; Weksler et al., 2013; Helms et al., 2016). It has also been reported that expression of endothelial tight junction specific *CLDN5* is significantly lower in immortalized human BMECS than *in vivo*. Taken together, BMECs originating from either animal or human tissue origins lose some of their organotypic phenotypes when cultured *in vitro* (Urich et al., 2012; Weksler et al., 2013). The use of all the previously mentioned brain endothelial cells in various mono- and co-culture conditions has highlighted the need for a stable *in vitro* BBB model possessing both vascular endothelial and barrier phenotypes (Helms et al., 2016).

## Human Pluripotent Stem Cell Differentiation as a Possible Alternative

Recently, Lippmann et al. (2012) have reported the generation of a pure population of putative BMECs from pluripotent stem cell sources (iBMECs) has been described to meet the need for a reliable and reproducible *in vitro* human BBB model. Human pluripotent stem cells, embryonic or induced, can differentiate into large quantities of specialized cells in order to study development and model disease. iBMECs are generated primarily through directed differentiation of pluripotent stem cells into both neural and endothelial progenitors followed by selective purification. Under these differentiation culture conditions, it is proposed that the neural cell types provide a microenvironmental cues that coax the emerging endothelial progenitors toward a BBB-specific phenotype as they further differentiate into ECs (Lippmann et al., 2012). Later iterations of this protocol reported that adding retinoic acid or inhibiting GSK3 during this neuro-endothelial differentiation process would enhance the yield and fidelity of these putative iBMECs (Lippmann et al., 2014; Qian et al., 2017; Faley et al., 2019). Additionally, others have developed a more defined serum-free method which aimed to improve the consistency of differentiated iBMECs while decreasing the overall length of the differentiation process (Hollmann et al., 2017; Neal et al., 2019) (Table 1). Regardless of the method used, these iBMECs display endothelial-like properties, such as tube formation and low-density lipoprotein uptake, high TEER (≥800 Ω.cm^2^), and barrier-like efflux transporter activities (Stebbins et al., 2016; Appelt-Menzel et al., 2017; Canfield et al., 2017; Hollmann et al., 2017; Lim et al., 2017; Stebbins Matthew et al., 2017; Vatine et al., 2017; Delsing et al., 2018; Sances et al., 2018). They have also been reported to express select BMEC marker transcripts such as *PECAM1, CDH5*, and *CLDN5*, among other BBB-specific markers (Qian et al., 2017; Vatine et al., 2017, 2019; Lee et al., 2018; Faley et al., 2019; Martins Gomes et al., 2019; Linville et al., 2020).

**TABLE 1:** Major iterations of hPSC-derived iBMEC protocols.

Year	References	Protocol	Changes vs. 2012	Cell line/Maintenance	Differentiation media	QPCR	Antibodies	Barrier assays	Transporters/transcytosis
2012	Lippmann et al. (2012). Nature biotechnology	(1) Prior diff cells are passaged on Matrigel (mTESR1 for 2–3 days. (2). Media is switched to lack of FGF (UM) for 5–7 days. (3). Switch to EC media human Endothelial Serum-Free Medium (Invitrogen) supplemented with 20 ng/mL bFGF and 1% platelet-poor plasma derived bovine serum32 (PDS; Biomedical Technologies, Inc.). (4) 1–2 days of EC medium treatment, cells were dissociated with dispase (2 mg/mL; Invitrogen) and plated onto 12-well tissue culture polystyrene plates and maintained in EC media.	NA	ES line: H9, IPS: IMR90-4, iPS-DF19-9-11T33, iPS-DF6-9-9T. Irradiated MEFS, DMEMF12 20%KOSR, 1xMEM, 1mM-lglutamine, 4ng.ml bFGF	UM = lack of FGF and EC = Endothelial Serum-Free Medium (Invitrogen) supplemented with 20 ng/mL bFGF and 1% platelet-poor plasma derived bovine serum32 (PDS; Biomedical Technologies, Inc.)	PECAM1, CDH5, vWF, LDLR, LRP1, INSR, LEPR, BCAM, TFRC, AGER, STRA6, SLC7A5, SLC1A1, SLC38A5, SLC16A1, SLC2A1, ABCB1, ABCG2, ABCC1, ABCC2, ABCC4, and ABCC5. PLVAP, SLC21A14, FST, FZD7, FZD4, FZD6, STRA6, LEF1, APCDD1, SLC2A1, ABCB1 control: GAPDH, NO EC CONTROL	PECAM-1 (Rabbit, Thermo Fisher) CLAUDIN-5 (Mouse, Invitrogen) Occludin (Mouse, Invitrogen) *P*-glycoprotein (Mouse, Thermo Fisher) GLUT-1a (Rabbit antiserum) VE-Cadherin (Mouse, SCBT) Nestin (Rabbit, Millipore) βIII tubulin (Rabbit, Sigma) βcatenin (FITC-conjugated Mouse, BD Biosciences) Wnt7a FISH Wnt7b FISH GFAP (Polyclonal Rabbit, Dako) aSMA (Mouse, American Research Products)	TEER, coculture with rat astrocytes	Inulin, sucrose, glucose, vincristine, colchicine, prazosin, diazepam, rodhamine 123 ((cyclosporine). No EC control
2014	Lippmann et al. (2014)Lippmann et al., . Scientific reports	(1) Prior diff cells are passaged on Matrigel (mTESR1 for 2–3 days. (2). Media is switched to lack of FGF (UM) for 5–7 days. (3). Switch to EC media human Endothelial Serum-Free Medium (Invitrogen) supplemented with 20 ng/mL bFGF and 1% platelet-poor plasma derived bovine serum32 (PDS; Biomedical Technologies, Inc.). (4). 1–2 days of EC medium treatment, cells were dissociated with dispase (2 mg/mL; Invitrogen) and plated onto 12-well tissue culture polystyrene plates and maintained in EC media (RA	Addition of Retinoic Acid on day 6 use of versene to dissociate the cells instead of dispase, results in less debris	IMR90-4 and DF19-9-11T iPSCs and H9 hESCs in mTESR or 2012	UM = lack of FGF and EC = Endothelial Serum-Free Medium (Invitrogen) supplemented with 20 ng/mL bFGF and 1% platelet-poor plasma derived bovine serum32 (PDS; Biomedical Technologies, Inc.) + RA	ABCB1, ABCG2, ABCC1, ABCC2, ABCC5, and STRA6	PECAM-1 (Rabbit, Thermo Scientific) GLUT-1 (Mouse, Thermo Scientific) Occludin (Mouse, Life Technologies) CLAUDIN-5 (Mouse, Life Technologies) VE-Cadherin (Mouse, SCBT) E-Cadherin (Goat, R&D Systems) *P*-glycoprotein (Mouse, Life Technologies) BCRP (Mouse, Millipore) MRP1 (Mouse, Millipore) GFAP (Rabbit, Dako) βIII tubulin (Rabbit, Sigma) Nestin (Mouse, Millipore) αSMA (Mouse, American Research Products) PDGFRβ (Rabbit, Cell Signaling)	TEER, coculture with NPC astrocytes, neurons and primary pericytes	DOXO, rhodamine DCFDA
2017	Qian et al. (2017). Science advances	6 (μM of CHIR on D0-1, he medium was removed and cells were transitioned to DeSR2 (DeSR1 plus B27 supplement) for another 5 days with daily medium changes. At day 6, cells were switched to hECSR1 medium [human endothelial serum-free medium (hESFM) supplemented with basic fibroblast growth factor (bFGF, 20 ng/ml), 10 (M RA, and B27] to induce RA signaling in the hPSC-derived endothelial progenitors in an attempt to drive the specification to BMECs. Cells were maintained in this medium for 2 days. At day 8, cells were replated onto a Matrigel-coated substrate in hECSR1, and at day 9, the medium was switched to hECSR2 (hECSR1 lacking RA and bFGF).	Accutase instead of Versene	Human iPSCs [iPS(IMR90)-4 (*72*), iPS-DF 19-9-11T (*73*), and hESCs (H9) (*29*)] were maintained on Matrigel (Corning)–coated surfaces in mTeSR1	6 μM CHIR99021 (Selleckchem) in DeSR1: DMEM/Ham’s F12 (Thermo Fisher Scientific), 1× MEM-NEAA (Thermo Fisher Scientific), 0.5× GlutaMAX (Thermo Fisher Scientific), and 0.1 mM β-mercaptoethanol (Sigma). After 24 h, the medium was changed to DeSR2: DeSR1 plus 1× B27 (Thermo Fisher Scientific) every day for another 5 days. At day 6, the medium was switched to hECSR1: hESFM (Thermo Fisher Scientific) supplemented with bFGF (20 ng/ml), 10 μM RA, and 1× B27		Brachyury (R&D Systems) PAX2 (SCBT) CD31 (Thermo Fisher) VE-Cadherin (SCBT) vWF (Dako) VEGFR2 (SCBT) CLAUDIN-5 (Invitrogen) Occludin (Invitrogen) ZO-1 (Invitrogen) GLUT-1 (Thermo Fisher) PGP (Thermo Fisher) BCRP (Millipore) MRP1 (Millipore) OCT3/4 (SCBT) TRA-1-60 (SCBT) NANOG (SCBT) ICAM-1 (R&D Systems)	“We also compared the differentiation reproducibility with that of the previously reported UM protocol (33). Although both methods produce BMECs capable of substantial barrier formation from multiple hPSC lines, BMECs differentiated from H9 hESCs and 19-9-11 iPSCs using the defined method exhibited higher TEERs and lower batch-to-batch variation.”	Efflux transporter activities were measured by the intracellular accumulation of (G) rhodamine 123, (H) Hoechst, and (I) DCFDA, substrates for Pgp, BCRP, and MRP, respectively. CsA, Ko143, and MK571 were used as specific inhibitors of Pgp, BCRP, and MRP, respectively
2017	Hollmann et al. (2017). Fluids barriers CNS	Modified 2014 protocol	E8 and E6 media, E6 for 4 days then continued as Lippmann et al. (2014) protocol.	MR90-4 iPSCs CC3 iPSCs, CD12 iPSCs, and SM14 iPSCs in growth factor-reduced Matrigel (VWR) in E8 medium	E8 medium was prepared by adding 100 μL of human insulin solution (Sigma-Aldrich), 500 μL of 10 mg/mL of human holo-transferrin (R&D Systems), 500 μL of 100 μg/mL human basic fibroblast growth factor (bFGF; PeproTech), and 500 μL of 2 μg/mL TGFβ1 (PeproTech) to 500 mL of E4. The final concentrations are 2.14 mg/L insulin, 100 μg/L bFGF, 2 μg/L TGFβ1, and 10.7 mg/L holo-transferrin E6 medium was prepared by adding 100 μL of human insulin solution and 500 μL of 10 mg/mL of human holo-transferrin to 500 mL of E4. The final concentrations are 2.14 mg/L insulin and 10.7 mg/L holo-transferrin UM and EC same as 2–14		PECAM-1 (Rabbit, Thermo Scientific) GLUT-1 (Mouse, Thermo Scientific) OCCLUDIN (Mouse, Thermo Scientific) CLAUDIN-5 (Mouse, Thermo Scientific) VE-Cadherin (Goat, R&D Systems) GFAP (Rabbit, Dako) PDGFR-B (Rabbit, SCBT) NG2 (Mouse, SCBT) αSMA (Mouse, SCBT)	TEER	Intracellular accumulation of rhodamine 123 (a Pgp substrate) was evaluated in the absence of bFGF and RA. Cells were incubated with 10 μM PSC833 or 10 μM MK-571 for 1 h at 37°C. They were then incubated for an additional house with 10 μM rhodamine 123 or 10 μM H2DCFDA.

**A summary of the original hPSC-derived iBMEC protocol (Lippmann et al., 2012) as well as the major adaptations made in subsequent studies (Lippmann et al., 2014; Hollmann et al., 2017; Qian et al., 2017) highlighting the key changes and experimental conditions used in each study*.*

iBMECs generated using the original neuro-endothelial differentiation and subsequent protocols have quickly been adopted as a robust and viable source of human BMECs in many different *in vitro* studies of the BBB. At their inception, iBMECs were primarily used as a monoculture system to recapitulate the BBB in two-dimensional cell culture conditions. The cells were described to replicate barrier and transporter phenotypes present in the BBB *in vivo* in a cell-autonomous manner as well as respond to signaling from other neural cell types and microenvironmental changes. iBMECs cocultured with astrocytes and/or pericytes have been reported to have increased TEER to above 1,500 Ω.cm^2^ as well as expression of certain transporters and receptors present in the BBB such as *SLC2A1*, *BCRP*, *MRP1*, and *LRP1* (Lippmann et al., 2012, 2014; Canfield et al., 2017; Qian et al., 2017; Vatine et al., 2017, 2019). Some groups have concluded that not only does the vascular endothelial identity of iBMECs remain stable in co-culture but that these conditions aid in the maturation of iBMECs into a more functional BBB model defined in large part by a decrease in dextran permeability through the barrier model.

Unsurprisingly, culturing these cells on a 2-D surface, regardless of the extra cellular matrix used, places observable limitations on cell–cell interactions including movement of secreted factors in the dish. These limitations would eventually lead to iBMECs being adapted to three-dimensional BBB models including various brain “organ-on-chip” models designed to have iBMECs interacting with various other cell types in 3-D (Sances et al., 2018; Faley et al., 2019). Some of these models also allow for flow to be introduced to the cells, further mimicking *in vivo* conditions (DeStefano et al., 2017; Vatine et al., 2019). Many groups have used 3-D iBMEC based models to further study the BBB under more physiologically relevant conditions; reporting data on permeability, gene expression, and barrier properties of iBMECs (Vatine et al., 2019; Linville et al., 2020). Over time, the use of both 2-D and 3-D iBMEC-based BBB models has led to many conclusions regarding the properties and functions of brain specific ECs which has added a lot of data to the field of BBB research. As a result of these reports, iBMECs have been widely accepted for use as a brain specific EC in many *in vitro* systems to assess BBB properties and function in homeostatic and disease models (Lippmann et al., 2012, 2013, 2014; Wilson et al., 2015; Stebbins et al., 2016; Appelt-Menzel et al., 2017; Canfield et al., 2017; Hollmann et al., 2017; Lim et al., 2017; Qian et al., 2017; Stebbins Matthew et al., 2017; Vatine et al., 2017, 2019; Delsing et al., 2018; Lee et al., 2018; Sances et al., 2018; Faley et al., 2019; Martins Gomes et al., 2019; Linville et al., 2020).

## Conflicting Reports of iBMEC Vascular Cell Identity

Cellular identity is multifaceted and is usually determined by a combination of transcriptional, translational and functional phenotypes presented by a cell. This concept is particularly important for PSC-derived cell types, as directed differentiation to a target cell may yield a heterogenous population where the target cell type is less frequent or lacking in canonical lineage-specific genes that can have a tremendous impact on the overall efficacy of the cell as an *in vitro* model. Moreover, there is an existing concern regarding the standards by which cells engineered *in vitro* are validated against their primary counterparts in native tissues (Daley, 2015). These concerns stem from reports using only a small number of transcriptional or protein markers, limited or peripheral functional assays, and global RNA expression analyses which taken together can lead to a misguided identification of the cell type produced *in vitro.*

The characterization of iBMECs has been dominated by their *in vitro* barrier properties which has largely been based off the TEER measurements of these cells in monolayer conditions (Stebbins et al., 2016). Expression of barrier specific genes such as *ZO-1* and *OCLN* have also served as a standard to bolster this barrier phenotype though these and many other tight junction marker genes expressed by iBMECs are not canonically specific to vascular ECs (Acharya et al., 2004; Hwang et al., 2013). Over time, some studies have started to present conflicting data regarding the cellular identity of these cells (Delsing et al., 2018; Lu et al., 2019; Vatine et al., 2019). Given that organ specific models rely heavily on cell specific responses to stimuli or cell–cell interactions which can differ widely depending on cell type, concerns over iBMEC cellular identity presents a major problem with their use in *in vitro* BBB models.

As the BBB is principally a vascular structure, it is imperative that iBMECs are phenotypically, transcriptionally and functionally analogous to definitive ECs which make up the BBB. Recently, there have been several reports demonstrating certain incongruencies in the cellular identity of iBMECs. Delsing et al. (2018) demonstrated that iBMECs generated from the neuro-endothelial differentiation expressed a considerably lower level of key endothelial marker genes in both mono and co-culture conditions when compared to other hPSC derived ECs or primary human BMECs. This study reported the presence of PECAM1, ZO-1, CLDN-5, VWF, and other endothelial markers in protein staining of iBMECs as well as hPSC derived ECs of a different protocol. However, the bulk RNA sequencing data reported a statistically significant decrease in mRNA expression of *PECAM1*, *CDH5*, *CLDN5*, and *VWF*, in iBMECs relative to the other ECs tested. The study went on to show that iBMECs express *CLDN4*, *CLDN6*, and *CLDN7* indicating the presence of an epithelial cell junction. Taken together, this data led the group to conclude that iBMECs possess somewhat of a mixed phenotype (Delsing et al., 2018).

In Lu et al. (2019), our group conducted an in-depth characterization of iBMECs using a combinatorial analysis of protein and RNA expression comparing iBMECs from previously published work to their own. Our analysis also included multiple primary endothelial cell controls as well as hPSC derived ECs generated using a another previously published protocol (James et al., 2010). Initial microscopy and fluorescence activated cell sorting (FACS) revealed a lack of PECAM1 and CDH5 protein expression in iBMECs compared to the other ECs used in this study. EPCAM protein expression is also demonstrated in exclusively the iBMECs using the same assays. To validate these results, we performed a meta-analysis of bulk RNA sequencing data comparing their own iBMECs to previously published RNA transcriptomes obtained from the NCBI Gene Expression Omnibus (GEO).

We were able to demonstrate that not only were their iBMECs transcriptionally equivalent with previously published iBMECs but also that all iBMECs lacked a canonical EC transcriptional profile and conversely expressed many genes normally related to an epithelial cell lineage such as *EPCAM*, *KRT8*, *KRT19*, *SPP1*, and *FREM2*. These results were confirmed by single-cell RNA sequencing which also showed iBMECs to be a homogenous epithelial cell population lacking a vascular EC identity. An absence of key EC transcription factor (TF) and marker genes was observed in both RNA sequencing platforms, concerns over the validity of the previously established protein expression data becomes apparent. iBMECs used in this study were shown to be transcriptionally identical to those previously published which mean that positive protein expression data could be due to non-specific binding by monoclonal antibodies, especially in the case of proteins from large homologous families such as Claudins (Krause et al., 2015). In summary, this study concludes that though iBMECs present a tight junction phenotype with high TEER, their cellular identity is severely lacking in congruency to vascular ECs making them unsuitable for use as an *in vitro* model of the human BBB (Lu et al., 2019) (Table 2).

**TABLE 2:** hPSC-derived iBMECs are not phenotypically comparable to primary human BMECs.

	hPSC-Derived BMEC *in vitro*	Primary Human BMEC *in vitro*
Surface marker profile	PECAM1^–^ CDH5^–^ EPCAM^+^	PECAM1^+^ CDH5^+^ EPCAM^–^
Claudin family repertoire	Claudin-4, Claudin-6, Claudin-7	Claudin-5
Barrier properties	High junctional electrical resistance	Low junctional electrical resistance
Inflammatory response	No canonical vascular response observed	VCAM-1, ICAM-2, E-Selectin upregulation
Significant media differences	Serum free (or 1% platelet poor bovine serum)	Fetal bovine serum; SB431542
Extracellular matrix	Fibronectin/collagen IV mixture	Gelatin

**An overview of crucial differences between hPSC-derived iBMECs and primary human BMECs illustrating major differences in cellular phenotype as well as in vitro culture conditions*.*

**FIGURE 1:**
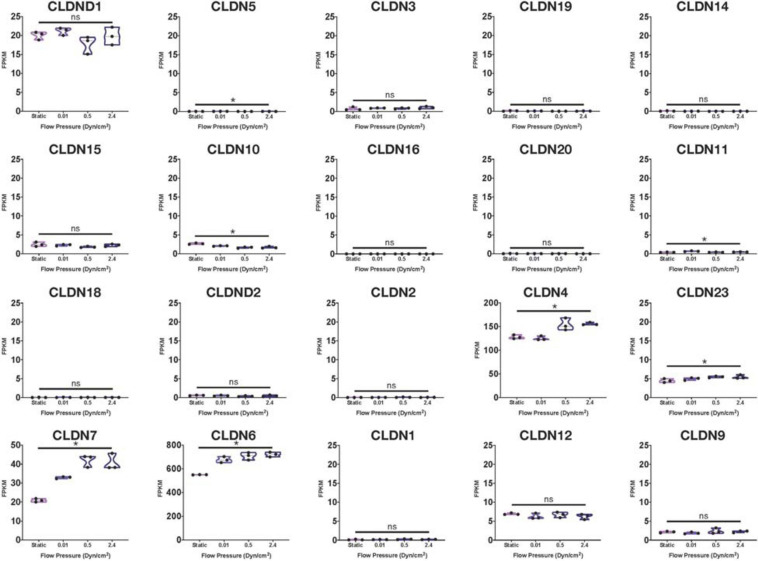
Claudin family RNA expression in iBMECs under sheer stress. Violin plots of Claudin family gene expression in iBMEC organ-chip samples at various flow pressures adapted from data provided by Vatine et al. (2019) (significance indicates *p*-value < 0.05).

**FIGURE 2:**
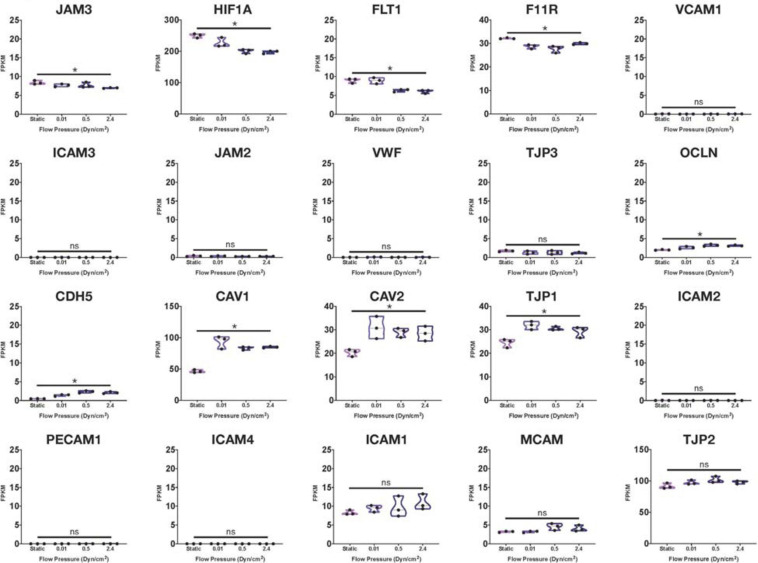
Junctional-related gene RNA expression in iBMECs under sheer stress. Violin plots of junctional-related gene expression in iBMEC organ-chip samples at various flow pressures adapted from data provided by Vatine et al. (2019). Genes were defined as junctional-related according to the referenced study (significance indicates *p*-value < 0.05).

Using many of the same bulk RNA endothelial and epithelial control samples from the GEO repository as Lu et al. (2019), and their own iBMECs, Vatine et al. (2019) performed a meta-analysis as well. iBMECs in this study were seeded in an organ-chip device in which the authors claim they establish a hollow vessel-like structure. A Principal Component Analysis (PCA) of their dataset revealed that iBMECs clustered closest to some of the endothelial controls. However, the lung epithelial cell libraries used in this analysis were prepared using total RNA without ribosomal depletion, while the rest of the dataset consisted of samples that were both polyA-primed and depleted of their ribosomal transcripts. Such a discrepancy in library preparation methods is likely to have caused a significant bias in this PCA; in fact, sample divergence across PC1 was exclusively due to the presence of ribosomal transcripts. Still, this meta-analysis also reported the presence of many epithelial cell transcripts in iBMEC samples, reinforcing previous conclusions about the presence of a non-vascular epithelial cell identity in these cells. This study goes on to introduce iBMEC organ-chips to different levels of laminar flow and co-culture with various hPSC-derived neural cell types. Bulk RNA sequencing of iBMECs in each of these conditions revealed a number of differences in expression of the *CLDN* gene family (Figure 1) as well as junction related genes (Figure 2) which the authors used to conclude that certain conditions allowed for the functional maturation of iBMECs (Vatine et al., 2019).

Importantly, these changes in expression levels were reported in relative terms for each transcript after normalization across samples that did not include an EC control. The FPKM values for some of the genes reported varied with statistical significance across conditions (*CLDN4*, *CLDN6*, *HIF1A*, and *CAV1*); however, some of these statistically significant differences denoted changes in FPKM values of less than 1 (*CLDN5*, *CLDN10*) stipulating an overall lack of function difference in gene expression. Interestingly, differences in FPKM values of greater than 20 occurs exclusively in genes more closely related to an epithelial lineage, reinforcing the notion of an epithelial phenotype in iBMECs. Differences in FPKM of some marker genes were also shown to be not significantly different between samples even though the Row *Z*-score demonstrates a large difference in expression. Moreover, the average FPKM of some of these marker genes across all samples was under 0.1 indicating that the genes are barely expressed and unlikely to undergo any translation (Hart et al., 2013). Interpretation of near zero FPKM values as functional expression of a gene could lead to incorrect assumptions of cell identity and functional phenotypes.

The lack of a functional vascular endothelial identity in iBMECs is further reinforced in a study by Martins Gomes et al. (2019) in their study focusing on their use as a disease model for *Neisseria meningitidis* (*Nm*) infection of the brain. While characterizing the response of iBMECs to inflammatory factors brought on by *Nm* infection, the group notes no difference in VCAM-1 or E-Selectin RNA expression which shows a lack of an EC specific response to inflammatory stimuli. Nishihara et al. (2020) further bolsters these results with their study in which they assess immune cell interactions with iBMECs. They characterized the inflammatory response of iBMECs generated with the established method (Lippmann et al., 2014) and those generated using the later adapted chemically defined method (Qian et al., 2017). Interestingly, it was shown that iBMECs differentiated using either method did not stain positive for ICAM-2, VCAM-1, E-selectin, or P-Selectin (Nishihara et al., 2020). ICAM-1 upregulation was only reported upon removal of retinoic acid, which was previously described to contribute greatly to the development of a vascular EC identity in these cells (Lippmann et al., 2014), from the differentiation process. The group ultimately concluded that iBMECs generated from any of these protocols lack expression of many vascular cell adhesion molecules and are not well suited for modeling immune cell interactions in the BBB.

## Possible Means for Induction of Vascular BBB Phenotype in hPSC-Derived Cells *In Vitro*

Before a functional hPSC-derived vascular BBB model can be developed, stable hPSC-derived vascular ECs must be generated. In a recent study (Lu et al., 2021), our group demonstrated that a vascular fate can be induced in iBMECs through introduction of certain EC-specific ETS TFs (*ETV2*, *ERG*, and *FLI1*). These reprogrammed cells (rECs) harbor an EC transcriptomic profile, retaining a PECAM1^+^CDH5^+^KDR^+^ EC immunophenotype during passaging and expansion. Purified rECs can respond to inflammatory stimuli (i.e., TNF-α) and permeabilizing agents (i.e., VEGF-A and anti-VE-cadherin antibody) in a manner congruent with vascular ECs. rECs were also shown to be capable of forming tubes *in vivo* using an immunocompromised mouse model, whereas iBMECs derived from the same hPSCs could not. This strategy of transcription factor reprogramming establishes a vascular EC identity in cells that otherwise lacked any phenotypic and functional aspects of bona fide ECs. However, further work is required to generate a reliable brain specific EC and that this only represents a crucial step toward the generation of true brain ECs suitable for *in vitro* modeling of physiological and pharmaceutical studies of the BBB.

In addition to the co-culture systems referenced above, numerous culture conditions have been demonstrated to improve BBB phenotypes *in vitro*. Some groups were able to show that neural cell conditioned media could increase barrier resistance and decrease permeability in BMEC monolayers (Siddharthan et al., 2007; Puech et al., 2018). Others have used cytokines and small molecules in the culture medium to modify barrier and vascular phenotypes these ECs. As previously mentioned, de Vries et al. (1996) demonstrated that exposure to cytokines such as TNF-α, IL1-β, and IL-6 induces an overall decline in TEER across rat brain EC monolayers. Schulze et al. (1997) later showed that Lysophosphatidic Acid increases tight junction permeability in porcine brain ECs. In contrast, a study from Roudnicky et al. (2020b) has indicated that the ALK5 inhibitor RepSox could modulate EC barrier stability. These studies all support the notion that microenvironmental queues play a large role in the homeostatic regulation of BMECs and adjustments to culture conditions will largely affect the overall function of an *in vitro* BBB model.

Moving on from culture conditions, intrinsic transcriptional regulation may also be critical for the establishment of a vascular BBB model. A separate Roudnicky et al. (2020a) study was able to demonstrate that synergistic overexpression of TFs including *SOX18*, *TAL1*, *SOX7*, and *ETS1* can enhance certain properties in EC such as barrier function. Their work shows that hPSC-derived ECs transduced with these TFs have increased transmembrane electrical resistance and tight junction protein expression while also decreasing paracellular transport (Roudnicky et al., 2020a). Taken together, this data suggests transcription factor overexpression could eventually be used in conjunction with chemomodulation in order to directly generate brain-specific ECs from hPSCs which could be suitable for *in vitro* BBB models.

## Concluding Remarks

Over the past decade many groups have aimed to advance the study of the BBB by developing *in vitro* models attempting to mimic the physiological complexity of the BBB *in vivo*. Many difficulties have arisen during the course of these efforts as such models must phenocopy the high TEER observed *in vivo* as well as the intricate cellular transport mechanisms that are hallmarks of the BBB. It has been demonstrated that BBB traits are not intrinsic to brain specific ECs, but rather the result of a dynamic interplay with their microenvironment including multiple cell types such as astrocytes and pericytes. Consequently, primary brain ECs lose their barrier properties, especially high TEER, when cultured *in vitro*. Many laboratories have attempted to resolve this issue by developing various *in vitro* BBB models using neural cell co-cultures consisting of ECs, pericytes, and astrocytes. These models also include pluripotent stem cell differentiation methods as well as brain organoids and ‘organ-on-a-chip’ approaches.

**FIGURE 3:**
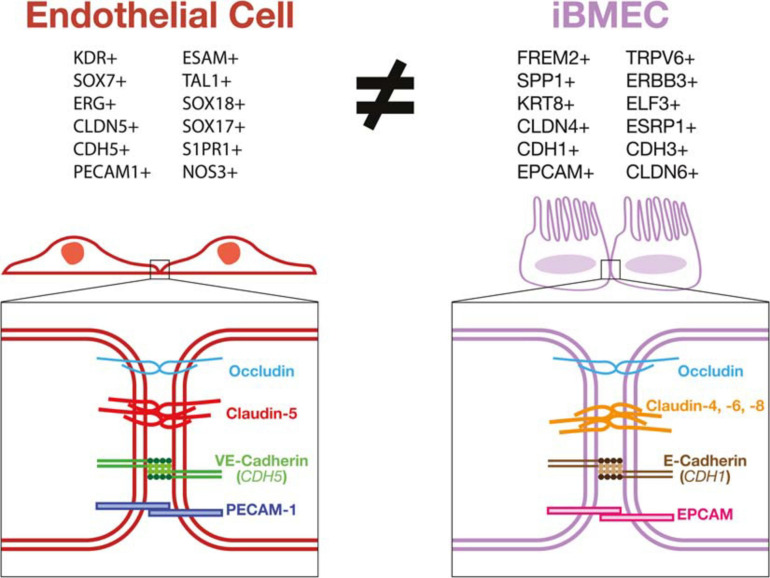
iBMECs do not possess an endothelial transcriptional profile or vascular junctional components. iBMECs are shown to lack expression of phenotypical markers of a vascular EC lineage while expressing many epithelial cell lineage genes by bulk and single-cell RNA sequencing methods. The junctional components in iBMECs are also incongruent with canonical EC junctions leading to very serious concerns as to the efficacy of their use in an *in vitro* vascular BBB model.

Validation of these *in vitro* models relies mainly on using TEER and expression of tight junction proteins as a determinant of barrier function. Using these measurements as a proxy for functional BBB-specific tight junctions presents some limitations since it can only measure the paracellular junctions. The BBB has many transcellular permeability functions that are imperative for its function which cannot be measured in this way. Additionally, high TEER and many of the junctional proteins used to validate brain EC identity have been demonstrated in other non-endothelial cell types such as epithelial cells. ECs have also been shown to possess a polarized morphology (Lizama and Zovein, 2013) similar to epithelial cells, however, these morphological and junctional characteristics do not suggest that epithelial cells can be used interchangeably with ECs in *in vitro* vascular barrier models. Other problems may also have arisen from assigning a vascular EC identity to hPSC-derived cells based on the expression of a restricted set of brain EC markers. False positive results of EC identity can occur in these cases due to antibody cross-reactivity with proteins present in the cell sample that are not specific to ECs. As shown, iBMECs may demonstrate a high TEER *in vitro* and express certain non-vascular specific junctional genes, however, they lack many functional phenotypes intrinsic to ECs. By not responding to inflammatory stimuli in an EC specific manner, their use in many models would yield results misrepresenting the *in vivo* BBB (Figure 3).

Taken altogether, the data presented by recent studies (Delsing et al., 2018; Lu et al., 2019; Martins Gomes et al., 2019; Vatine et al., 2019; Nishihara et al., 2020) contradict the vascular cellular identity of iBMECs and instead demonstrate that these cells might be of an epithelial lineage. iBMECs have been shown to lack expression of key EC marker genes such as *PECAM1*, *CDH5*, *CLDN5*, and *VWF* while also expressing epithelial cell genes including *EPCAM*, *FREM2*, and *CLDN4*. Expression of genes such as E-Selectin, VCAM-1, P-Selectin were also shown to be completely unaffected by inflammatory stimuli further decreasing the possibility for these cells to be used as a functional model of the BBB *in vitro*. This leads to the possibility that the barrier function observed in iBMECs could in fact more closely resemble an epithelial cell barrier such as the choroid plexus or intestinal epithelial barrier. As these cells lack a canonical vascular EC phenotype, the use of current protocols to generate iBMECs as prototypical human BBB model could results in inaccurate physiological studies and screening for misguided druggable targets or treatments with potential ineffective clinical outcomes. Thus, the application of a rigorous and thorough characterization of stem cell-derived products using the latest available technologies such as single cell multi-omics and metabolomics should be necessary, rather than facultative, for the development of faithful disease models and safe cell-based therapies.

## Data Availability Statement

The original contributions generated for this study are included in the article/supplementary material, further inquiries can be directed to the corresponding authors.

## Author Contributions

All authors listed have made a substantial, direct and intellectual contribution to the work, and approved it for publication.

## Conflict of Interest

The authors declare that the research was conducted in the absence of any commercial or financial relationships that could be construed as a potential conflict of interest.
